# Approach to evaluate the sensory quality deterioration of chicken seasoning using characteristic oxidation indicators

**DOI:** 10.1016/j.fochx.2023.100564

**Published:** 2023-01-07

**Authors:** Hao-Yu Xu, Xiao-Wei Chen, Jun Li, Yan-Lan Bi

**Affiliations:** a Lipid Technology and Engineering, College of Food Science and Engineering, Henan University of Technology, Lianhua Road 100, Zhengzhou 450001, Henan Province, PR China; b Food Laboratory of Zhongyuan, Henan University of Technology, Zhengzhou 450001, PR China

**Keywords:** Chicken seasoning, Sensory quality, Lipid oxidation, GC–MS, Oxidation indicators

## Abstract

Sensory quality deterioration of chicken seasoning was investigated using physicochemical properties, gas chromatography-mass spectrometry (GC–MS) and descriptive sensory analysis to approach an evaluation of the chicken seasoning deterioration. It was found that both peroxide value (POV) and total oxidation value (TOTOX) increased with the chicken seasoning deterioration, suggesting a dominant of the lipid oxidation in the sensory quality deterioration of chicken seasoning. Moreover, a continuously decreasing linoleic acid and contradictory increasing in volatile aldehydes (specifically for hexanal) indicated as characteristic oxidation indicators to evaluate the sensory quality deterioration. PLSR results further elucidated that the evolution of aldehydes was highly correlated with sensory quality deterioration. These results suggest the POV, TOTOX and hexanal as valuable indicators and provide a novel approach to quality and rapidly evaluate the sensory quality deterioration of chicken seasoning.

## Highlights

• Oil oxidation is the dominant factor in the quality deterioration of chicken seasoning.
• POV and TOTOX could evaluate quantically the deterioration of chicken seasoning.
• Multiple techniques evaluated aldehydes were highly correlated with sensory profiles.
• Volatile hexanal as indicators for evaluating the deterioration of chicken essence seasoning.

## Graphical abstract

**Figure:**
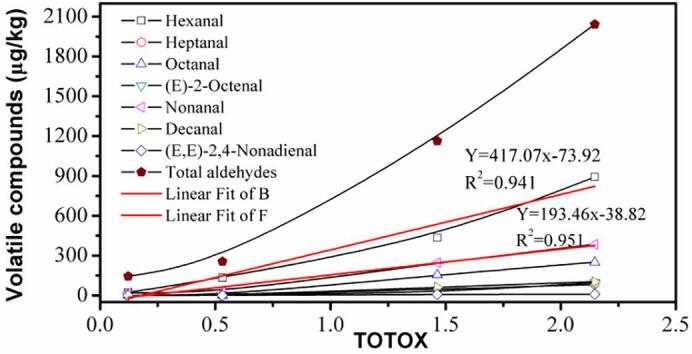

## Introduction

Chicken seasoning has been widely used as an important food ingredient in China to improve food flavor. It not only presents the umami advantage of ordinary monosodium glutamate, but also inherits the essence of traditional Chinese compound seasoning broth with the natural flavor of chicken (Zhu et al., 2020). Over the next five years, the market for chicken seasoning in China is expected to grow 17.19 % annually and reached 495 million. Unfortunately, it is prone to sensory quality deterioration during storage (Beltran et al., 2003). A particularly important attribute when it comes to choosing seasonings is the sensory quality, which is traditionally assessed by a descriptive sensory analysis.

Human sensory evaluation has been employed for the determination of the deterioration in chicken seasoning. For example, Tian et al. (2014) used sensory evaluation to study the sensory quality deterioration and found 8 sensory attributes. The sensory evaluation provides integrated, direct measurements of perceived intensities of target attributes (Fang et al., 2019, Tian et al., 2019). However, it is time-consuming and easily influenced by a trained taster's mental and physical state. Recently, electronic nose has been developed for the evaluation of many tastes (Ali et al., 2020, Hu and Jacobsen, 2016). By simulating the human sense of taste, the electronic tongue helps differentiate samples and formulations (Tian et al., 2014). Although it can give good prediction results for the sensory attributes, an expensive taste sensor is necessity (Tian et al., 2014, Tian et al., 2018). Moreover, it is still unknown how the sensory quality of chicken seasoning deteriorates during storage. To get more specific information, chemical analysis is expected to apply due to a good repeatability and credibility.

Recently, some chemical technologies like GC–MS have been employed to assess the sensory quality deterioration of seasonings. Tian et al. (2018) detected fourteen compounds including aldehydes, sulfurs, ketones, and heterocyclic compounds using GC–MS, and found those compounds are associated with an increase in lipid oxidation. Beltran et al. (2003) discovered that one of the most important factors influencing the sensory quality of cooked and pressurized chicken is lipid oxidation. Lipids in chicken seasoning are important to the nutrition and flavor; however, they are prone to oxidation because of their high polyunsaturated fatty acids (PUFA) content. Therefore, the lipid components of chicken seasoning undergo oxidation during storage, which seems to negatively impact their nutritional value and quality (Cao et al., 2014, Tian et al., 2019). Nonetheless, there is non-objective approach to evaluate the sensory quality deterioration of chicken seasoning using characteristic oxidation indicators. Additionally, there are no clear relationships among sensory descriptors, physicochemical properties, and volatile compounds identified by chicken seasoning discrimination to explore usable and sensitive oxidation indicators for evaluating the sensory quality deterioration of chicken seasoning.

The principal objectives of this study were to (a) determine whether oil oxidation is the cause of sensory deterioration of chicken seasonings by observing the changes of physicochemical indexes, fatty acid composition and volatile substances, (b) to investigate the relationship between fat composition and volatile substances of chicken seasonings by principal component analysis (PCA), (c) and to investigate the relationship between sensory properties and volatile substances of chicken seasonings by multivariate statistical techniques including hierarchical clustering analysis (HCA), pearson correlation coefficient (PCCs) and partial least-squares regression (PLSR) to clarify the characteristic oxidation indicators in the sensory deterioration of chicken seasonings. The findings would provide good and sensitive indicators for the quality and rapid evaluation of chicken seasoning deterioration.

## Materials and methods

### Chemicals and materials

Commercial chicken seasoning (78 packages, a certain brand with the same ingredients, containing sodium glutamate, salt, sugar, rice flour, chicken, whole egg liquid, flavors, onion, garlic, disodium 5′-ribonucleotide and riboflavin) were purchased from dozens supermarkets in Zhengzhou and Shanghai, and the deterioration was determined by sensory evaluation and divided into different levels of deterioration based on the intensity of rancid flavor, where 30 packs of fresh ones (F), 9 packs of light deteriorated ones (L), 16 packs of medium deteriorated ones (M) and 23 packs of serious deteriorated ones (S). 2,4,6-trimethylpyridine, the mixed standards of 37 kinds of fatty acid methyl esters and C7-C40 saturated alkanes were purchased from Sigma-Aldrich Co. (St. Louis, MO, USA). All the other chemical reagents were of analytical grade.

### Determination of crude fat and moisture content

Both indicators were determined according to AOCS official methods Ba 3-38 and Ba 2a-38.

### Determination of oil oxidation

#### Peroxidation value.

The peroxide value (POV) was determined referring to the report by Jensen et al. (2011) with minor modifications. First, 1.0 g of crushed chicken seasoning was mixed evenly with 5 mL of trichloromethane/methanol mixture (7:3, v/v) solution, followed by centrifuging for 2 min at 4000*g*. Next, the supernatant was transferred into a flask, followed by diluting to 10 mL with trichloromethane/methanol (7:3, v/v), and adding 50 μL of ferric chloride solution (3.5 g/L) and 50 μL of potassium thiocyanate solutions (300 g/L). Finally, the reaction kept still for 5 min under dark conditions before measuring the absorbance at 500 nm by an UV–vis spectrophotometer (TU-1810, Beijing Purkinj General Instrument Co., ltd).

#### Total oxidation value.

Total oxidation value (TOTOX) is commonly selected because it is an effective oxidative indicator or marker for determining the entire primary and secondary lipid oxidation product. For bulk oils, TOTOX is suitable to measure POV and anisidine value. However, conventional TOTOX values could not be used to evaluate complicated multiphase food systems, such as anhydrous foods. According to the report by Hu and Jacobsen (2016), a widely used TOTOX value could be defined as: $\mathrm{TOTOX}=4\times POV+100\times \sum \frac{C_{n}}{1000\times M_{n}}$, where POV is peroxide value (mmol/kg); C_n_ is the content of aldehydes (μg/kg); Mn is the molar mass of an aldehyde (g/mol); 100: conversion factor; 1000: content conversion factor.

### Fatty acids composition analysis

The composition of fatty acids was analyzed using gas chromatography (GC). Firstly, the oil in chicken seasoning was extracted and methylated as described by Yang et al. (2021). The fatty acid methyl esters (FAMEs) were analyzed using an 8860 GC (Agilent Technologies, USA) equipped with a capillary column (SGE BPX-70, 30 m × 250 μm × 0.25 μm) (SGE Analytical Science, UAS). The injector and detector temperatures were both set at 250 °C. The oven temperature was set at 170 °C and then increased to 210 °C at a rate of 2 °C/min. The flow rate of N_2_ carrier gas was set at 1.0 mL/min. Each component was identified by corresponding retention time compared to the mixed standards of 37 kinds of FAMEs, and quantified by the area normalization method.

### SPME-GC–Ms

The volatile compounds in chicken seasoning were analyzed using a solid-phase microextraction gas chromatography-mass spectrometry approach (SPME-GC–MS). Firstly, a 15-mL vial was filled with 5.0 g of chicken seasoning and 5.0 g of water. 30 μL of internal standard (2,4,6-trimethylpyridine, 65 μg/g) was added and incubated in a thermostat water bath at 65 °C for 30 min. Then, a solid-phase microextraction fiber (SPME, 65 μm PDMS/DVB, Supelco, Sigma Aldrich) was used to enrich the volatile compounds for 30 min. Then, the volatile compounds were measured using an 7890A GC equipped with a HP-5MS capillary column (30 m × 255 μm × 0.25 μm, Agilent Technologies, USA) and an Agilent 5975C mass detector. The oven temperature was held at 50 °C for 5 min, then increased to 90 °C at 2 °C/min and maintained for 5 min, followed by climbing to 230 °C at 10 °C/min and maintained for 5 min. Mass spectrometry analysis was performed under a full scan mode with a *m*/*z* range of 33–500. Ionization was accomplished using electron impact (EI) at a source temperature of 230 °C and an electronic energy of 70 eV. Each compound was identified by retention index (RI) and mass spectrometry (MS), and quantified by internal standard method. RI meant that the compounds were identified by comparing with literature data <25, MS meant that the compounds were identified by comparing mass spectra in the NIST11.L library search system with a match of more than 80 %.

### Sensory evaluation

Quantitative descriptive analysis (QDA) was used to evaluate sensory attributes using a ten-point hedonic scale, where a score of 10 indicated an excellent product and a score of 1 indicated an absolute poor product. The sensory panel consisted of twelve assessors (trained scientists and post graduate students of the division, six male and six female) aging from 21 to 54 years old, who were selected and trained based on the Sensory Evaluation Practices proposed by Stone et al. (2012). Panelists were briefed about the nature of experiments and technically cultivated for 6 h to guarantee the sensory descriptors of chicken seasoning including rancidity, chicken flavor, fishy smell, fat, umami, whole aroma and acceptability (Tian et al., 2018). All participants acknowledged informed consent prior to this study, and the rights and privacy of each participant were ensured. All sensory sessions were conducted in individual booths in a test room designed to meet the requirements of ISO 8589:2007. 30 g of each chicken seasoning was placed in a brown wide-mouth bottle and sealed in a 65 °C water bath for 5 min before being presented to the sensory panelists. All samples were individually numbered in three-digital numbers at random and evaluated one after the other using QDA. Compusense Cloud (Compusense Inc., Ontario, Canada) was used to collect sensory data.

### Statistical analysis

All experiments were carried out at least twice, and data were presented as mean ± standard deviation. Principal component analysis (PCA) and partial least squares regression (PLSR) were statistically analyzed using The Unscrambler X (ver. 10.4, CAMO software, Norway), hieratical cluster analysis (HCA) and Pearson correlation coefficient (PCCs) were analyzed and visualized using R studio (ver. 3.6.3, AT&T Bell Laboratories, USA). The rest of the data was analyzed by Duncan’s multiple range tests using SPSS (ver. 26.0, IBM SPSS, USA) and plotted by Origin Pro (ver. 2022, OriginLab, USA).

## Results and discussion

### Quality changes of chicken seasoning

**Fig. 1:**
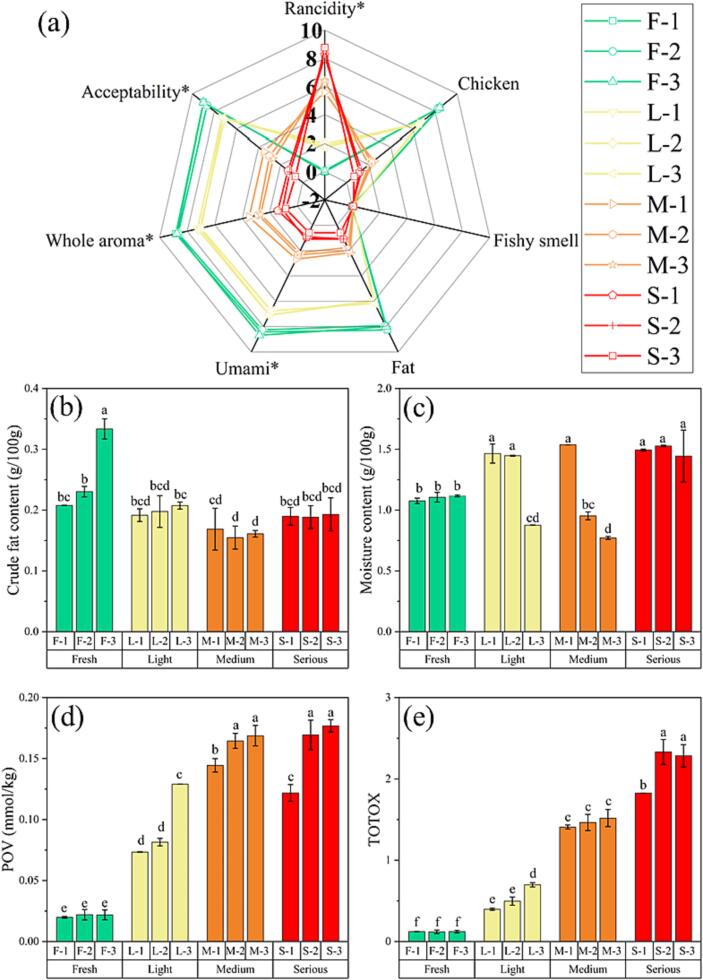
(a) The radar map of the chicken seasonings, where the * in the graph of flavor indicate there was a significant difference between the four groups of samples (*p* < 0.05). Crude fat content (b), moisture content (c), POV (d) and TOTOX (e) of chicken seasoning as function of the deterioration levels form sensory evaluation.

As illustrated in Fig. 1, the quality of chicken seasoning was evaluated by sensory, fat content, moisture content, peroxide value (POV), and total oxidation value (TOTOX). Typical chicken seasonings were investigated, such as three fresh ones (F), three light deteriorate ones (L), three medium deteriorate ones (M) and three serious deteriorate ones (S). A radar map was based on the score of the sensory attributes of three groups with different storage times. From Fig. 1**a**, obvious differences in the four groups were observed for rancidity, chicken flavor, fat, umami, whole aroma and acceptability; that is, the characteristic flavor of chicken seasoning deteriorated as a result of sensory quality degradation (Tian et al., 2014). Additionally, rancidity scores were ascended significantly as storage time, which indicates that the samples developed an unpleasant flavor during the storage. Tian et al. (2018) previously reported that the deteriorated chicken seasoning has a rancid flavor. Meanwhile, Sohn & Ohshima, (2010) also found that the rancid flavor of raw fish is produced by lipid oxidation.

Lipid oxidation is a major cause of quality degradation in oil-rich foods, resulting in a decrease in nutritional and sensory value (Ghorbani Gorji et al., 2019). Thus, the fat content, moisture content, POV and TOTOX were evaluated. From Fig. 1**b**, low crude oil was observed in these chicken seasonings ranging from 0.15 to 0.33 g/100 g with an average value of 0.20 g/100 g. The slight drop in oil content with increased storage time might be attributed to its loss in the form of oxidation products (Tian et al., 2014). Moreover, there was no obvious change in the moisture content from 0.77 to 1.54 g/100 g with an average value of 1.23 g/100 g during the storage of the four groups (Fig. 1**c**). Focusing on the physical properties only, there was not a regular trend, suggesting both oil and moisture content cannot be used to evaluate the deterioration of chicken seasoning. Similar results had been reported by Tian et al. (2014). However, the physical properties of this low-fat and low-moisture content provided the basis for the selection of a method to determine the peroxide value.

In spite of low oil content, it was considered to be an important influence on the quality deterioration of chicken seasoning (Tian et al., 2018). As demonstrated in Fig. 1**d**, there was a significant (*p* < 0.05) increase in POV as chicken seasoning deterioration intensified, where from 0.020 to 0.022 mmol/kg for the F group, 0.073–0.129 mmol/kg for the L group, 0.144–0.169 mmol/kg for the M group to 0.122–0.177 mmol/kg for the S group. The POV of the S group was expanded about 6–9 times compared with the F group, indicating an oil oxidation in the chicken seasoning during storage. TOTOX was commonly used to assess the degree of oil oxidation (Esfarjani et al., 2019). From Fig. 1**e**, TOTOX raised from an initial level of 0.12 ± 0.00 to 2.33 ± 0.15 after the sensory quality deterioration of chicken seasoning. Moreover, the order of oxidation degree of the four groups of samples was: group S > group M > group L > group F. These results indicated oil oxidation was the main factor in chicken seasoning deterioration, and both POV and TOTOX were key indicators for evaluating the oxidation status of the chicken seasoning.

### Changes of the fatty acid composition

**Fig. 2:**
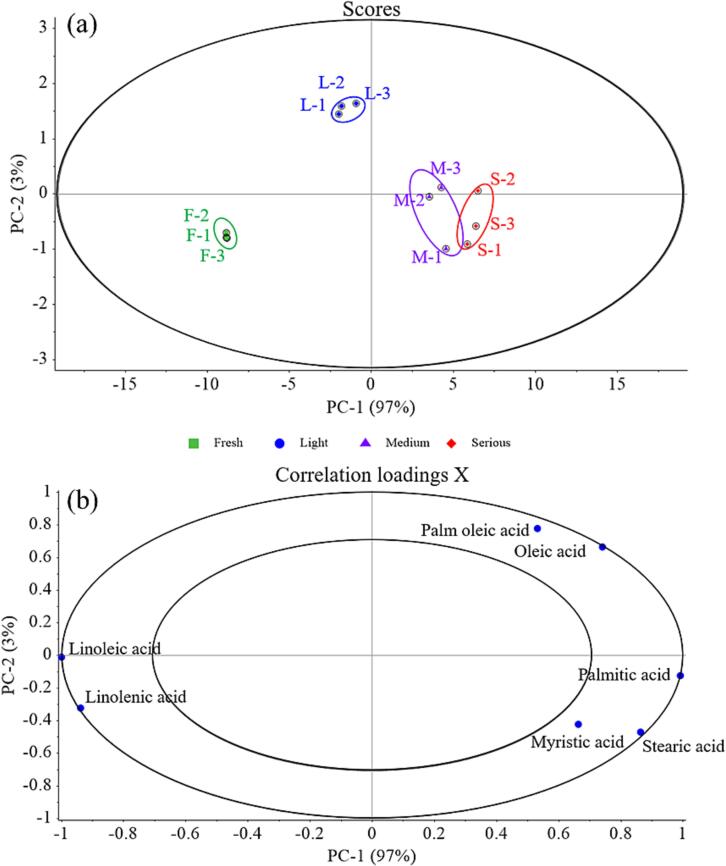
Principal component analysis (PCA) score plot (a) and loading plot (b) of fatty acid composition in chicken seasoning samples.

The fatty acid composition of oil in chicken seasoning was also determined, as shown in Table 1. A total of 8 fatty acids were identified in the fat of chicken seasoning. There were mostly unsaturated fatty acids, such as oleic acid and linoleic acid, with around 4 % palm oleic acid. The unsaturated fatty acid content showed a decreasing significantly (*p* < 0.05) with the deterioration increasing. For example, the highest linoleic acid (20.07 %-25.05 %) and linolenic acid (1.39 % to 1.93 %) were observed in the F ones, but the lowest linoleic acid (11.82 %–12.26 %) and linolenic acid (0.65 % to 0.91 %) were observed in the S ones. To insight the relationship between the fatty acid composition and sensory quality deterioration, PCA was further performed, as shown in Fig. 2. From the Fig. 2**a**, the contribution of PC1 was 97 % while PC2 contributed 3 %, showing that the recovered principal component factors may accurately capture the vast majority of information from the original data (Tian et al., 2014, Tian et al., 2018). Moreover, the projection of the four groups on the PC_1_ axis has no intersection, which indicates that PC_1_ is the largest contributor to distinguish the four groups from each other. The loading plot depicts the coefficients of the independent variable (fatty acids), and the larger the absolute value of the load coordinate, the greater the effect on the principal components (Cui et al., 2020). It can be seen from Fig. 2**b** that the PC_1_ coefficients of fatty acids (*e.g.*, linoleic acid, linolenic acid, oleic acid, palmitic acid and stearic acid) were greater than that of PC_2_ coefficients, indicating PC_1_ reflects mainly the information of these five fatty acids. Meanwhile, the negative values of linoleic acid and linolenic on the PC_1_ coordinates were observed, suggesting a negative relationship; while as oleic acid, palmitic acid and stearic acid was confirmed positively correlated with PC_1_. These results can be claimed that the linoleic acid was severely oxidized during the deterioration of chicken seasoning, followed by linolenic acid.

**Table 1:** Fatty acid composition (%) in fat of chicken seasoning.a.

Fatty acids	Samples											
	F1	F2	F3	L1	L2	L3	M1	M2	M3	S1	S2	S3
Myristic acid	0.54 ± 0.00^f^	0.54 ± 0.00^f^	0.54 ± 0.00^f^	0.60 ± 0.00^de^	0.59 ± 0.00^e^	0.61 ± 0.01^d^	0.66 ± 0.01^bc^	0.67 ± 0.00^bc^	0.66 ± 0.00^c^	0.69 ± 0.01^a^	0.69 ± 0.00^a^	0.68 ± 0.02^ab^
Palmitic acid	24.80 ± 0.02^f^	24.76 ± 0.05^f^	24.85 ± 0.01^f^	27.05 ± 0.19^e^	27.24 ± 0.03^de^	27.65 ± 0.12^d^	31.08 ± 0.03^bc^	30.63 ± 0.07^c^	30.81 ± 0.04^c^	31.56 ± 0.22^ab^	31.81 ± 0.02^a^	31.95 ± 0.65^ab^
Palm oleic acid	3.58 ± 0.01^c^	3.61 ± 0.04^c^	3.54 ± 0.00^c^	4.62 ± 0.68^ab^	5.19 ± 0.01^a^	5.03 ± 0.04^a^	3.80 ± 0.06^c^	4.74 ± 0.08^a^	4.77 ± 0.00^a^	4.01 ± 0.66^bc^	4.82 ± 0.05^a^	4.74 ± 0.05^a^
Stearic acid	5.49 ± 0.01^d^	5.44 ± 0.01^d^	5.53 ± 0.04^d^	5.44 ± 0.09^d^	5.47 ± 0.05^d^	5.57 ± 0.00^d^	6.78 ± 0.03^a^	6.16 ± 0.02^c^	6.26 ± 0.01^bc^	6.62 ± 0.18^a^	6.41 ± 0.02^b^	6.42 ± 0.15^b^
Oleic acid	38.08 ± 0.02^d^	38.16 ± 0.03^d^	38.14 ± 0.06^d^	40.85 ± 0.52^a^	40.78 ± 0.09^a^	40.83 ± 0.07^a^	40.02 ± 0.07^c^	40.37 ± 0.09^bc^	40.6 ± 0.09^ab^	40.12 ± 0.07^c^	40.86 ± 0.09^a^	40.15 ± 0.06^c^
Linoleic acid	25.04 ± 0.02^a^	25.05 ± 0.02^a^	25.02 ± 0.02^a^	18.93 ± 0.38^b^	18.87 ± 0.05^b^	18.11 ± 0.13^b^	13.52 ± 0.12^d^	14.48 ± 0.04^c^	13.78 ± 0.00^cd^	12.26 ± 0.01^e^	11.82 ± 0.02^e^	11.91 ± 1.13^e^
Linolenic acid	1.86 ± 0.11^a^	1.93 ± 0.00^a^	1.93 ± 0.01^a^	1.02 ± 0.07^b^	1.01 ± 0.01^b^	0.91 ± 0.02^b^	0.79 ± 0.05^c^	0.92 ± 0.01^b^	0.76 ± 0.01^cd^	0.91 ± 0.02^b^	0.65 ± 0.00^d^	0.73 ± 0.07^cd^
Others	0.61 ± 0.03^f^	0.50 ± 0.15^f^	0.45 ± 0.08^f^	1.50 ± 0.00^e^	0.85 ± 0.07^f^	1.29 ± 0.26^e^	3.35 ± 0.09^bc^	2.03 ± 0.28^d^	2.36 ± 0.04^d^	3.84 ± 0.15^a^	2.94 ± 0.10^c^	3.42 ± 0.40^ab^
MUFAs	41.66 ± 0.03^g^	41.77 ± 0.07^g^	41.68 ± 0.06^g^	45.47 ± 0.16^bcd^	45.97 ± 0.09^a^	45.86 ± 0.02^ab^	43.82 ± 0.00^f^	45.11 ± 0.16^de^	45.37 ± 0.09^cd^	44.12 ± 0.59^f^	45.68 ± 0.04^abc^	44.89 ± 0.02^e^
PUFAs	26.90 ± 0.09^a^	26.99 ± 0.02^a^	26.95 ± 0.03^a^	19.95 ± 0.45^b^	19.88 ± 0.06^bc^	19.02 ± 0.15^c^	14.31 ± 0.17^e^	15.40 ± 0.03^d^	14.54 ± 0.01^de^	13.18 ± 0.03^f^	12.47 ± 0.03^f^	12.64 ± 1.20^f^
UFAs	68.56 ± 0.06^a^	68.76 ± 0.09^a^	68.63 ± 0.10^a^	65.42 ± 0.29^b^	65.85 ± 0.15^b^	64.88 ± 0.13^b^	58.13 ± 0.17^d^	60.52 ± 0.19^c^	59.91 ± 0.10^c^	57.30 ± 0.56^d^	58.15 ± 0.07^d^	57.53 ± 1.22^d^
SFAs	30.83 ± 0.03^e^	30.74 ± 0.06^e^	30.92 ± 0.02^e^	33.08 ± 0.29^d^	33.3 ± 0.08^cd^	33.83 ± 0.13^c^	38.52 ± 0.08^a^	37.46 ± 0.09^b^	37.73 ± 0.05^b^	38.86 ± 0.40^a^	38.91 ± 0.04^a^	39.05 ± 0.82^a^

a MUFAs: monounsaturated fatty acids, PUFAs: polyunsaturated fatty acids, UFAs: unsaturated fatty acids, SFAs: Saturated fatty acids.; Different superscript letters represent significant differences with other values in the same row at p < 0.05.

### Changes of the volatile compounds

The sensory quality deterioration of chicken seasoning was more noticeably in volatile compounds. Further, the volatile compounds in these chicken seasonings were quantified using GC–MS. A total of 55 volatile compounds were identified, including 3 sulfurs, 13 aldehydes, 10 ketones, 4 alcohols and phenols, 11 olefins, 2 alkanes, 2 heterocycles, 5 pyrazines and 5 esters From the Tables 2, six aldehydes were detected in the fresh samples. Moreover, the content of these aldehydes increased sharply (*p* < 0.05) with the increase of deterioration, such as hexanal and nonanal from 18.46 to 25.89 μg/kg and 12.49–25.56 μg/kg to 711.05–995.49 μg/kg and 329.39–366.76 μg/kg, respectively. It was worth noted that some new aldehydes were detected in the deteriorated samples, such as heptanal, octanal, (E)-2-octenal, decanal, (E, E)-2,4-nonadienal, (E)-2-decenal, and 2-butyl-2-octenal. As known, aldehydes are the typical secondary oxidation products resulting from oil oxidation, where both hexanal and heptanal are produced by the oxidation of linoleic acid; (E)-2-octenal is produced by the oxidation of linoleic acid to produce 2,4 decadienal through a reverse alcohol-aldol condensation reaction; while octanal, nonanal, decanal, and (E)-2-decenal are produced by the oxidation of oleic acid (Cao et al., 2014, Cao et al., 2020, Kalua et al., 2007, Multari et al., 2019). The increase in hexanal, heptanal, and (E)-2-octenal was correlated with a decrease in linoleic acid content in the deteriorated samples (Table 1). The elevated content of octanal, nonanal and decanal indicates that the oxidation of oleic acid also occurred during the deterioration of chicken seasoning, which is consistent with some previous studies where the contents of hexanal, heptanal, octanal, nonanal and pentanal increased in chicken, beef and pork that underwent lipid oxidation after irradiation (Kwon et al., 2008, Zheng et al., 2022). More importantly, the odor thresholds of these aldehydes are usually low and have important effects on the flavor of foods (Chen et al., 2016). For example, hexanal (5 μg/kg) and nonanal (1.1 μg/kg), which impart a green, fatty flavor to foods at low concentrations, but cause rancid flavor at high concentrations (Brewer et al., 1999, Van, 2011). This finding was also consistent with the results for the oil oxidation (Fig. 1**d and 1e**). Therefore, it is confirmed that these aldehydes are responsible for the rancid flavor of chicken seasoning. Although the contents of alkanes and olefins increased from 2.39–6.32 μg/kg and 148.16–295.56 μg/kg to 7.79–18.49 μg/kg and 361.88–628.23 μg/kg, respectively, the odor thresholds of these two types of volatiles are usually high and often play a basal role for the flavor in foods (Liu et al., 2021, Tian et al., 2019).

**Table 2:** Characteristic volatile compounds (μg/kg) in different chicken seasonings.

**No**	**Compounds**	**RI**^a^	**RIcal*^b^***	**ID*^c^***	**Threshold** **(μg/kg)*^d^***	**Aroma description *^e^***	**Samples**											
							**F1**	**F2**	**F3**	**L1**	**L2**	**L3**	**M1**	**M2**	**M3**	**S1**	**S2**	**S3**
1	Methyl furfuryl disulfide	1230	1229	RI, MS	0.04	smoking, fumigation	8.03 ± 0.62^d^	5.14 ± 0.94^e^	4.45 ± 0.63^ef^	2.90 ± 0.39^f^	3.82 ± 1.29^ef^	ND	5.53 ± 0.06^e^	10.47 ± 0.95^c^	5.30 ± 1.33^e^	15.25 ± 1.22^a^	9.00 ± 0.92^cd^	12.87 ± 0.66^b^
2	Diallyl trisulfide	1294		MS		garlic	91.17 ± 0.10^a^	31.93 ± 9.48^ef^	26.45 ± 5.71^f^	13.24 ± 0.34^g^	24.20 ± 2.47^fg^	12.69 ± 4.24^g^	38.44 ± 5.11^de^	50.71 ± 1.54^bc^	59.78 ± 4.02^b^	23.48 ± 0.96^fg^	86.40 ± 9.60^a^	43.93 ± 0.42^cd^
3	Difurfuryl dsulfide	1687		MS	0.00015	nutty, coffee, meaty	12.70 ± 5.63^ab^	10.87 ± 4.50^ab^	7.58 ± 0.17^ab^	5.29 ± 0.33^ab^	3.27 ± 2.38^b^	4.74 ± 2.03^b^	13.74 ± 10.49^ab^	7.13 ± 1.46^ab^	7.69 ± 2.93^ab^	13.63 ± 0.78^ab^	16.65 ± 4.99^a^	7.10 ± 1.97^ab^
4	Hexanal	799	801	RI, MS	5	green, fatty	24.81 ± 0.90^e^	18.46 ± 2.58^e^	25.89 ± 0.21^e^	84.22 ± 13.68^de^	139.49 ± 34.65^de^	173.67 ± 44.44^d^	449.70 ± 14.47^c^	447.35 ± 55.16^c^	406.86 ± 32.76^c^	711.05 ± 26.79^b^	970.46 ± 100.58^a^	995.49 ± 154.15^a^
5	Heptanal	902	903	RI, MS	2.8	citrus-like,green	ND	ND	ND	4.62 ± 0.36^c^	6.24 ± 2.39^c^	7.92 ± 2.77^c^	44.02 ± 2.77^b^	44.23 ± 7.38^b^	48.12 ± 6.91^b^	78.53 ± 3.11^a^	79.17 ± 9.42^a^	86.35 ± 14.25^a^
6	2-Methyl-2-pentenal	829		MS	290	strawberry, fruity	9.15 ± 0.62^a^	6.07 ± 1.30^b^	4.76 ± 0.38^c^	ND	ND	ND	ND	ND	ND	ND	ND	ND
7	Benzaldehyde	968	960	RI, MS	751	sweet, almond, caramel	15.73 ± 0.25^cde^	13.60 ± 2.86^de^	12.32 ± 0.39^e^	23.30 ± 2.12^a^	18.95 ± 3.73^abc^	22.66 ± 1.89^ab^	19.27 ± 0.01^abc^	15.31 ± 2.37^cde^	18.29 ± 2.34^bcd^	21.02 ± 1.66^ab^	19.09 ± 1.68^abc^	17.88 ± 1.40^bcd^
8	Octanal	1019	1006	RI, MS	0.59	fatty, soapy, green	ND	ND	ND	ND	ND	ND	155.36 ± 12.40^b^	140.90 ± 8.91^b^	164.02 ± 18.83^b^	253.20 ± 5.64^a^	258.70 ± 37.28^a^	239.60 ± 8.27^a^
9	(E)-2-Octenal	1079	1060	RI, MS	3	green, nutty, fatty	ND	ND	ND	5.44 ± 0.17^e^	7.02 ± 0.35^e^	8.31 ± 3.57^e^	15.01 ± 0.25^d^	18.36 ± 1.44^cd^	23.71 ± 3.31^b^	22.80 ± 0.46^bc^	29.46 ± 4.51^a^	32.15 ± 0.20^a^
10	Nonanal	1130	1104	MS	1.1	fatty, citrus-like, green	25.56 ± 1.27^f^	18.64 ± 2.96^f^	12.49 ± 0.72^f^	16.05 ± 0.18^f^	24.97 ± 3.58^f^	19.62 ± 3.60^f^	229.29 ± 21.96^de^	224.15 ± 10.70^e^	284.94 ± 22.76^cd^	366.76 ± 1.76^b^	456.81 ± 69.99^a^	329.39 ± 13.08^bc^
11	Decanal	1233	1209	RI, MS	3	soapy, orange peel, butter	ND	ND	ND	ND	3.13 ± 0.39^d^	ND	65.95 ± 8.31^c^	54.00 ± 1.32^c^	64.94 ± 4.08^c^	127.84 ± 8.41^a^	97.58 ± 14.38^b^	85.63 ± 11.29^b^
12	(E,E)-2,4-Nonadienal	1239	1217	RI, MS	0.1	fatty, waxy, green	ND	ND	ND	ND	1.57 ± 1.04^d^	ND	9.34 ± 0.35^b^	6.32 ± 0.75^c^	8.49 ± 2.46^bc^	6.94 ± 0.55^c^	10.74 ± 0.45^abc^	11.66 ± 1.38^a^
13	(E)-2-Decenal	1283	1262	RI, MS	17	citrus-like	ND	ND	ND	4.81 ± 0.11^e^	4.76 ± 1.37^e^	6.75 ± 1.67^e^	34.88 ± 3.92^d^	39.84 ± 1.56^d^	52.61 ± 6.01^bc^	84.57 ± 4.60^a^	42.08 ± 2.12^cd^	62.79 ± 11.80^b^
14	(E)-innamaldehyde	1286	1283	RI, MS	750	cinnamon, spicy	78.51 ± 10.94^bc^	72.45 ± 15.77^bcde^	67.23 ± 3.04^bcde^	36.19 ± 2.38^e^	50.94 ± 11.30^cde^	40.73 ± 10.91^de^	62.61 ± 43.12^bcde^	78.04 ± 3.46^bcd^	65.76 ± 2.06^bcde^	121.74 ± 8.53^a^	96.83 ± 3.25^ab^	116.71 ± 14.49^a^
15	(E,E)-2,4-Decadienal	1344	1317	MS	0.027	fried, chicken fat	9.56 ± 0.08^ef^	7.77 ± 1.44^f^	8.99 ± 0.42^ef^	17.07 ± 1.30^cd^	14.86 ± 0.20^de^	23.92 ± 6.89^ab^	21.08 ± 0.82^bc^	16.82 ± 0.20^cd^	17.07 ± 0.20^cd^	29.76 ± 1.89^a^	16.72 ± 2.60^cd^	21.10 ± 3.84^bc^
16	2-Butyl-2-octenal	1381		MS			ND	ND	ND	ND	ND	ND	79.14 ± 8.82^b^	32.25 ± 1.16^c^	33.44 ± 9.34^c^	147.33 ± 18.01^a^	32.35 ± 4.34^cd^	47.19 ± 9.14^c^
17	2-Heptanone	891	895	RI, MS	140	soapy, fruity	ND	ND	ND	3.66 ± 1.01^ef^	ND	5.33 ± 2.11^de^	14.17 ± 0.17^b^	7.86 ± 0.97^cde^	8.73 ± 0.51^cd^	22.03 ± 5.01^a^	11.04 ± 1.46^bc^	22.42 ± 2.32^a^
18	Dihydrothiophen-3(2H)-one	957		MS		creamy, onion, garlic	51.70 ± 0.07^a^	45.23 ± 9.63^ab^	39.34 ± 0.59^bcd^	43.19 ± 4.51^abc^	30.26 ± 3.97^def^	38.01 ± 1.28^bcd^	32.53 ± 0.24^cdef^	28.05 ± 3.59^cdef^	26.23 ± 0.78^ef^	25.21 ± 4.52^ef^	33.64 ± 5.54^cde^	21.88 ± 8.22^f^
19	3-Octene-2-one	1060	1040	RI, MS	6.7	nutty	ND	ND	ND	9.84 ± 2.04^de^	13.18 ± 2.57^d^	11.06 ± 2.17^de^	95.87 ± 5.09^b^	54.91 ± 5.31^c^	60.91 ± 4.48^c^	140.69 ± 0.54^a^	88.45 ± 10.44^b^	147.32 ± 8.63^a^
20	(E,E)-3,5-Octadien-2-one	1093		MS	100	fruity, fatty, mushroom	ND	ND	9.69 ± 0.53^ab^	10.28 ± 0.36^ab^	11.65 ± 0.31^a^	8.07 ± 4.05^bc^	5.75 ± 0.96^c^	5.73 ± 0.07^c^	5.66 ± 0.36^c^	ND	6.31 ± 0.60^c^	ND
21	3,5-Octadien-2-one	1117	1095	RI, MS			ND	ND	8.55 ± 0.81^c^	17.78 ± 1.54^a^	17.71 ± 2.45^a^	13.96 ± 6.28^ab^	13.10 ± 0.25^abc^	12.47 ± 0.61^abc^	10.43 ± 0.4^bc^	12.04 ± 2.77^bc^	11.98 ± 0.67^bc^	12.65 ± 0.83^abc^
22	Ethanone	1205		MS	21	bitter almond	ND	3.54 ± 0.55^d^	5.13 ± 2.07^d^	8.85 ± 0.23^c^	8.32 ± 1.64^c^	10.67 ± 2.48^bc^	15.81 ± 0.46^a^	10.99 ± 0.14^bc^	9.45 ± 2.07^c^	13.19 ± 0.30^ab^	10.77 ± 0.14^bc^	11.01 ± 0.93^bc^
23	Ar-Tumerone	1670		MS			448.33 ± 82.26^abcd^	346.59 ± 12.26^cdef^	373.43 ± 28.39^bcde^	298.23 ± 42.21^efg^	214.52 ± 35.51^g^	239.27 ± 20.70^fg^	513.69 ± 114.08^a^	466.60 ± 5.33^abc^	389.80 ± 52.72^bcde^	538.32 ± 25.29^a^	469.43 ± 13.12^ab^	339.08 ± 48.66^def^
24	Tumerone	1674		MS			362.85 ± 79.60^a^	262.99 ± 4.39^b^	145.27 ± 13.84^c^	63.23 ± 6.76^de^	46.41 ± 23.27^e^	45.52 ± 0.36^e^	131.73 ± 28.87^cd^	111.30 ± 2.69^cde^	95.20 ± 28.91^cde^	99.42 ± 0.45^cde^	164.88 ± 35.64^c^	70.48 ± 24.97^de^
25	Germacrone	1699		MS			8.60 ± 3.07^cde^	6.86 ± 0.80^de^	6.82 ± 1.14^de^	6.15 ± 0.65^de^	3.81 ± 1.10^e^	4.32 ± 0.25^e^	12.89 ± 5.30^bc^	7.91 ± 0.73^cde^	8.79 ± 1.23^cde^	19.39 ± 2.62^a^	14.60 ± 1.81^ab^	11.32 ± 2.23^bcd^
26	Curlone	1705		MS			221.5 ± 45.05^a^	171.80 ± 2.66^abc^	140.55 ± 13.08^bcd^	105.81 ± 16.47^def^	70.14 ± 15.28^f^	78.81 ± 9.94^ef^	155.11 ± 39.55^bcd^	158.49 ± 2.52^bcd^	128.93 ± 19.32^cde^	187.60 ± 5.36^ab^	152.67 ± 7.22^bcd^	130.26 ± 30.44^cde^
27	Eucalyptol	1045	1030	RI, MS	1.1	mint, sweet	17.55 ± 0.88^bc^	13.52 ± 2.18^c^	15.57 ± 1.43^c^	15.18 ± 2.43^c^	16.27 ± 2.67^c^	18.56 ± 6.08^bc^	19.16 ± 4.29^bc^	27.98 ± 2.09^a^	30.43 ± 0.97^ab^	27.51 ± 6.88^a^	29.30 ± 0.01^a^	24.69 ± 1.71^ab^
28	Linolool	1125	1100	RI, MS	6	floral, lavender	127.83 ± 9.54^b^	92.13 ± 8.03^cd^	124.45 ± 20.06^bc^	80.94 ± 6.42^d^	81.09 ± 3.23^d^	76.18 ± 34.17^d^	136.68 ± 2.84^b^	133.73 ± 4.64^b^	152.87 ± 1.48^ab^	124.72 ± 1.61^bc^	178.40 ± 16.98^a^	130.43 ± 6.88^b^
29	4-Terpineol	1197	1179	RI, MS	1200	turpentine, nutmeg	12.70 ± 1.92^ab^	8.78 ± 0.23^b^	11.74 ± 0.56^ab^	9.80 ± 1.17^ab^	10.96 ± 1.13^ab^	12.41 ± 4.76^ab^	9.83 ± 0.40^ab^	14.62 ± 0.35^a^	14.93 ± 0.49^a^	8.98 ± 0.17^b^	13.60 ± 2.62^ab^	12.29 ± 3.32^ab^
30	Ethyl Maltol	1221		MS		sweet, bready	102.36 ± 15.55^bcd^	88.57 ± 20.62^de^	107.90 ± 2.19^bcd^	85.48 ± 0.95^de^	82.35 ± 28.68^de^	63.64 ± 1.02^e^	134.76 ± 23.13^ab^	101.36 ± 3.17^bcd^	94.76 ± 3.65^cde^	147.46 ± 14.98^a^	115.26 ± 14.88^abcd^	127.84 ± 2.33^abc^
31	Limonene	1043		MS	200	lemon, citrus-like	7.39 ± 0.82^ab^	5.34 ± 1.22^b^	6.93 ± 0.68^ab^	7.53 ± 1.14^ab^	5.09 ± 1.54^b^	6.33 ± 2.72^b^	6.12 ± 2.97^b^	10.77 ± 0.83^a^	7.38 ± 0.55^ab^	9.09 ± 2.08^ab^	8.73 ± 0.90^ab^	8.49 ± 0.11^ab^
32	(-)-β-Caryophyllene	1408	1467	MS	64	woody, irritating	8.06 ± 1.86^de^	7.04 ± 1.06^e^	7.80 ± 1.53^de^	6.46 ± 0.85^e^	7.39 ± 0.35^de^	4.85 ± 0.60^e^	25.70 ± 4.33^a^	15.43 ± 0.37^bc^	12.73 ± 4.19^cd^	27.51 ± 3.57^a^	18.34 ± 2.08^b^	17.73 ± 0.53^bc^
33	(1E,4E,8E)-α-Humulene	1448	1467	RI, MS	160	woody	7.45 ± 0.73^cde^	5.50 ± 0.76^ef^	5.69 ± 1.04^def^	3.43 ± 0.43^fg^	4.23 ± 0.11^fg^	2.87 ± 0.05^g^	10.98 ± 1.70^ab^	8.55 ± 0.10^bc^	8.20 ± 2.24^cd^	11.65 ± 1.11^a^	8.85 ± 0.81^bc^	8.21 ± 0.72^cd^
34	(E)-β-farnesene	1457	1391	MS			3.55 ± 0.70^cd^	2.66 ± 0.65^d^	2.55 ± 1.52^d^	2.87 ± 0.59^d^	2.49 ± 0.26^d^	1.52 ± 0.50^d^	7.08 ± 1.10^ab^	5.98 ± 0.03^b^	6.10 ± 0.92^b^	8.46 ± 1.40^a^	5.74 ± 1.13^b^	5.62 ± 0.50^bc^
35	(Z)-β-Farnesene	1460	1445	RI, MS			2.37 ± 0.53^def^	1.90 ± 0.22^f^	ND	1.49 ± 0.33^f^	2.03 ± 0.01^ef^	ND	9.42 ± 1.93^b^	5.82 ± 0.01^c^	4.62 ± 2.28^cde^	16.70 ± 1.87^a^	4.66 ± 0.86^cd^	6.15 ± 0.45^c^
36	β-Curcumene	1479		MS			4.34 ± 1.85^de^	4.00 ± 0.03^e^	4.22 ± 1.01^e^	3.55 ± 0.35^e^	3.40 ± 0.41^e^	2.77 ± 0.38^e^	11.72 ± 1.92^a^	9.37 ± 0.48^abc^	7.86 ± 2.85^bc^	10.63 ± 1.27^ab^	10.19 ± 0.17^abc^	7.36 ± 0.30^cd^
37	α-Curcumene	1483	1553	MS			47.04 ± 9.46^c^	38.37 ± 8.57^c^	54.95 ± 14.99^c^	52.24 ± 4.92^c^	51.11 ± 3.32^c^	41.49 ± 12.66^c^	188.37 ± 34.34^a^	125.54 ± 1.22^b^	106.25 ± 41.20^b^	191.35 ± 22.40^a^	136.90 ± 9.13^b^	108.12 ± 0.42^b^
38	α-Zingiberene	1494	1494	RI, MS			77.43 ± 28.13^abc^	68.18 ± 5.39^bc^	105.50 ± 0.47^a^	19.51 ± 1.57^de^	21.54 ± 4.88^de^	15.20 ± 3.90^e^	96.82 ± 15.85^ab^	57.50 ± 0.50^c^	50.93 ± 26.44^cd^	68.26 ± 12.25^ab^	77.59 ± 16.99^abc^	50.46 ± 6.13^cd^
39	β-bisabolene	1507	1498	RI, MS			18.93 ± 7.73^d^	18.22 ± 2.09^d^	22.45 ± 4.09^cd^	17.09 ± 0.82^d^	18.44 ± 0.40^d^	15.17 ± 3.68^d^	61.63 ± 11.13^a^	38.64 ± 0.14^b^	33.03 ± 12.06^bc^	62.49 ± 2.68^a^	43.76 ± 2.00^b^	35.58 ± 0.91^bc^
40	β-Sesquiphellandrene	1523	1560	MS			90.44 ± 22.86^defg^	69.85 ± 14.55^fg^	81.68 ± 17.67^efg^	62.85 ± 5.76^fg^	60.25 ± 2.13^fg^	51.81 ± 14.91^g^	194.80 ± 36.90^ab^	140.84 ± 1.80^bcd^	113.48 ± 47.82^cdef^	211.56 ± 26.50^a^	155.06 ± 12.44^bc^	130.98 ± 10.31^cde^
41	a-Farnesene	1532	1500	MS			3.60 ± 0.51^cd^	2.87 ± 0.14^cde^	3.77 ± 1.25^cd^	2.99 ± 0.73^cd^	ND	2.42 ± 0.26^de^	7.79 ± 1.64^ab^	4.34 ± 0.10^cd^	5.61 ± 0.45^ab^	10.53 ± 2.84^a^	7.69 ± 0.68^ab^	5.63 ± 1.81^ab^
42	Anethole	1277		MS	50	fennel, sweet	610.65 ± 33.56^cd^	381.75 ± 34.11^e^	515.68 ± 47.15^d^	322.46 ± 9.89^ef^	286.24 ± 33.38^ef^	247.91 ± 63.95^f^	635.75 ± 66.80^c^	623.06 ± 0.75^c^	660.44 ± 11.67^bc^	763.02 ± 36.59^bc^	842.13 ± 80.26^a^	667.14 ± 0.45^bc^
43	Caryophyllene oxide	1582	1573	RI, MS	410	vanilla, sweet	11.00 ± 2.68^bcd^	8.16 ± 0.89^bcd^	10.72 ± 1.70^bcd^	7.65 ± 0.85^bcd^	4.83 ± 2.08^d^	3.42 ± 0.23^d^	15.57 ± 3.40^ab^	8.19 ± 0.66^bcd^	7.39 ± 1.50^cd^	21.10 ± 8.15^a^	12.98 ± 5.03^bc^	9.38 ± 0.19^bcd^
44	3-Methylpentadecane	1572		MS			ND	ND	ND	3.51 ± 0.37^cd^	1.97 ± 0.28^d^	ND	7.12 ± 1.39^b^	4.24 ± 0.56^c^	4.27 ± 1.41^c^	10.76 ± 0.87^a^	6.94 ± 0.29^b^	8.88 ± 1.52^ab^
45	Hexadecane	1599	1600	RI, MS	500	alkanes	2.94 ± 0.74^d^	2.23 ± 0.19^d^	4.71 ± 1.58^d^	2.81 ± 0.31^d^	2.96 ± 0.13^d^	2.39 ± 0.47^d^	13.12 ± 2.77^b^	7.83 ± 0.71^c^	7.79 ± 2.08^c^	18.49 ± 0.78^a^	10.35 ± 0.31^bc^	9.16 ± 0.65^c^
46	2,6-Dimethylpyrazine	914	913	RI, MS	718	bakery, cocoa	19.08 ± 0.40^d^	17.67 ± 3.26^d^	13.51 ± 2.22^d^	50.42 ± 0.53^b^	30.42 ± 7.29^c^	30.27 ± 6.77^c^	ND	16.95 ± 5.51^d^	32.29 ± 4.16^c^	72.45 ± 5.62^a^	12.39 ± 0.53^d^	ND
47	2,3,5-Trimethylpyrazine	1015	1000	RI, MS	350	bakery, nutty	58.39 ± 0.85^cd^	56.91 ± 2.76^def^	64.91 ± 5.46^c^	85.38 ± 5.06^a^	67.95 ± 7.90^c^	84.37 ± 4.67^ab^	38.20 ± 10.41^f^	33.94 ± 3.70^f^	69.76 ± 9.58^bc^	42.39 ± 9.11^ef^	36.98 ± 0.68^f^	48.70 ± 1.47^def^
48	Acetylpyrazine	1038	1023	RI, MS	60	bakery	28.22 ± 0.52^ab^	27.99 ± 5.74^ab^	25.19 ± 0.78^bc^	35.07 ± 2.73^a^	28.30 ± 2.18^ab^	27.75 ± 6.86^b^	24.03 ± 0.21^bc^	21.49 ± 1.11^bc^	21.22 ± 0.24^bc^	21.08 ± 0.39^bc^	20.08 ± 1.89^c^	21.45 ± 2.16^bc^
49	2,5-Diethyl-3-methylpyrazine	1111		MS	8.6	roasted potatoes	3.35 ± 1.46^cd^	3.43 ± 1.14^cd^	ND	16.09 ± 0.04^b^	ND	15.02 ± 5.19^b^	ND	6.12 ± 0.49^c^	16.80 ± 1.74^ab^	ND	21.07 ± 2.38^a^	ND
50	2-Methyl-3-methylsulfanylpyrazine	1187		MS	4		20.37 ± 0.66^f^	18.28 ± 1.76^f^	24.90 ± 0.10^e^	20.20 ± 0.57^f^	ND	ND	ND	31.51 ± 1.03^d^	42.18 ± 1.02^a^	38.54 ± 1.24^b^	34.64 ± 2.07^cd^	35.24 ± 3.60^bc^
51	2-Isocyanatooxane	1170		MS			ND	ND	65.07 ± 0.92^a^	5.96 ± 2.66^de^	7.36 ± 4.32^d^	19.61 ± 3.49^c^	14.71 ± 0.26^c^	19.62 ± 0.32^c^	15.62 ± 2.58^c^	32.16 ± 1.45^b^	17.62 ± 2.59^c^	29.82 ± 6.92^b^
52	γ-Octalactone	1278	1261	RI, MS	7.95	coconut	ND	ND	ND	ND	ND	ND	14.05 ± 0.95^b^	8.76 ± 0.58^c^	8.27 ± 1.39^c^	20.35 ± 2.42^a^	9.43 ± 0.32^c^	18.75 ± 4.41^a^
53	γ-Nonalactone	1371	1366	RI, MS	65		ND	ND	ND	ND	ND	ND	14.92 ± 1.70^b^	8.35 ± 0.17^c^	7.59 ± 1.11^c^	24.15 ± 2.25^a^	9.10 ± 1.17^c^	20.90 ± 6.01^a^
54	Triacetin	1374		MS			151.20 ± 53.92^bcde^	94.53 ± 14.83^e^	109.65 ± 13.48^de^	106.57 ± 9.28^e^	142.77 ± 50.56^bcde^	82.75 ± 11.62^e^	143.93 ± 37.26^bcde^	192.84 ± 22.3^abc^	212.52 ± 38.29^ab^	179.33 ± 22.12^abcd^	245.38 ± 13.58^a^	135.60 ± 5.75^cde^
55	Geranyl acetate	1390	1382	RI, MS	150	rose, lavender	ND	ND	ND	3.02 ± 0.41^c^	3.92 ± 0.12^c^	2.54 ± 0.22^c^	19.67 ± 2.91^a^	13.98 ± 0.02^b^	14.35 ± 3.00^b^	10.83 ± 3.52^b^	19.56 ± 0.50^a^	12.51 ± 1.68^b^

a RI was the calculated retention index. *^b^*RIcal and *^e^*Aroma descriptions were available from the Flavornet and human odor space (Terry Acree & Heinrich Arn, http://flavornet.org/index.html). *^c^*ID was the identification method. *^d^*Threshold value of compounds were available from a literature (Van G, L. J. 2011). ND not detected. Different superscript letters represent significant differences with other values in the same row at p < 0.05.

**Fig. 3:**
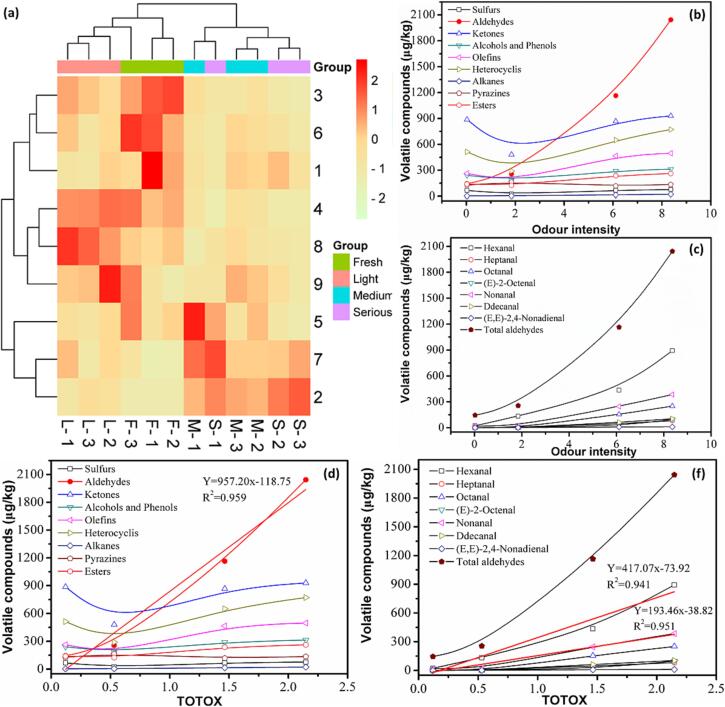
(a) Heatmap analysis results of various volatile compounds in chicken seasoning. Rows represent compound classes and columns represent samples. 1: sulfur compounds, 2: aldehydes, 3: ketones, 4: alcohols and phenols, 5: olefins, 6: heterocyclic compounds, 7: alkanes, 8: pyrazines, 9: esters. Relationship of the volatile compounds (μg/kg chicken seasoning) and the odor intensity (b, c) and TOTOX (d, f).

The volatile compounds were further processed as a cluster heat map, as shown in Fig. 3**a**. The heatmap's color coding progressed from green to red, while the concentration of aroma compounds went from low (green) to high (red). Longitudinally, the four groups of samples were divided into two major categories, fresh and light deteriorated samples were divided into one category, medium and serious deteriorated samples were divided into the other category. The phenomenon indicated that the volatile compounds contents of fresh and light deteriorated samples were similar, while the medium and serious deteriorated samples were corresponding to aldehydes and alkanes (Liu et al., 2021). Horizontally, aldehydes, alkanes, and olefins were divided into one category and other compounds were divided into another category. It revealed that the concentration of various volatiles differed significantly between deteriorated and fresh samples, where unfriendly flavor (*e.g.,* aldehydes, olefins and alkanes) were higher in medium and serious deteriorated ones. Similar findings have been reported in a variety of foods (Gayoso et al., 2017, Ghorbani Gorji et al., 2019, Ma et al., 2019). Moreover, as shown in Fig. 3**b**, it is very obvious that chicken seasoning had their characteristic aldehydes compounds, which could be used as an important marker for the chicken seasoning. In these samples with high linoleic acid levels, hexanal was the most abundant aldehyde, followed by nonanal and octanal (Fig. 3**c**). As shown in Fig. 3**d**, good correlations (R^2^ = 0.959) were observed between the total aldehydes amount (y) and TOTOX (x). Among the aldehydes, first-order kinetic equation between nonpolar hexanal (R^2^ = 0.941) and nonanal (R^2^ = 0.951) amount and TOTOX during lipid oxidation of the chicken seasoning (Fig. 3**d**), showing that the amount of aldehydes (particularly hexanal) in chicken seasoning might be utilized to predict oxidative deterioration as TOTOX. Similar findings were reported in many additional studies on edible oils and oil-rich foods (Cao et al., 2014, Chen et al., 2022).

### Relationship between volatile compounds and sensory properties

**Fig. 4:**
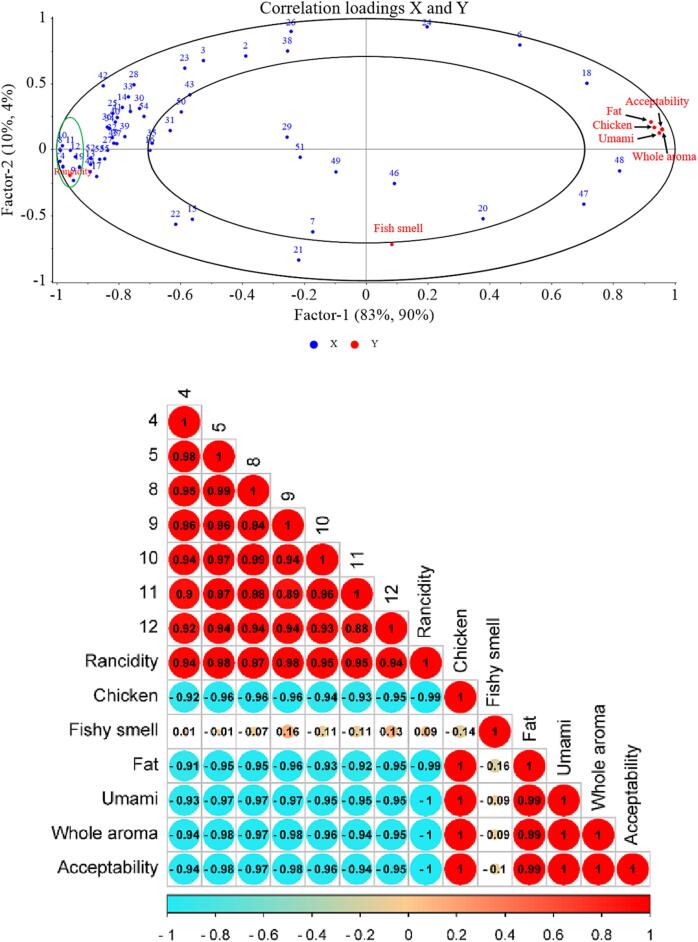
(a) Analysis of volatile compounds and sensory properties of the four sample groups by PLSR; Code of the volatile compounds correspond to those in Table 2. (b) Heat map of PCCs between seven aldehydes and sensory properties. The compounds in the small oval on the left are, 4: hexanal, 5: heptanal, 8: octanal, 9: (E)-2-octenal, 10: nonanal, 11: decanal, 12: (E, E)-2,4-nonadienal.

The PLSR results provide further information on the sensory attributes of 55 volatile compounds. From Fig. 4**a**, the PLSR modelling between selected volatile and sensory attributes was presented in a two-factor model elucidating 83 % and 10 % of the difference in X (designated volatiles) and in Y (sensory attributes) respectively, with a cumulative variance contribution of 93 %, indicating that most of the information of the samples can be successfully reflected by this PLSR model (Tian et al., 2014). The fishy smell was placed below the Factor 2 axis and was not surrounded by loads, indicating that the assessed aroma compounds could not explain all of the sensory attributes. It corresponds to the sensory profile of the samples (Fig. 1**a**), where the fishy smell was close to 0 for all four groups. Except for the fishy, the rancid flavor was separated from the rest of the sensory properties on both sides of the Factor1 axis, indicating that rancidity was negatively correlated to chicken aroma, fat aroma, umami aroma, whole aroma and acceptability. The seven aldehydes located in the small oval on the left are the ones with the largest Factor1 axis coordinates and the closest distance to the rancid flavor, indicating that these seven aldehydes were positively correlated with rancid flavor and their increased content was responsible for the rancid flavor of the chicken seasoning. In this case, the PCCs findings had strong correlation coefficient (≥0.9) for the prediction of the seven aldehydes and the unfavorable taste and overall acceptability (Fig. 4**b**), demonstrating that aldehydes had a significant relationship with all sensory attributes. Similar results were also found in investigating the effects of natural ingredients on the shelf life of chicken seasoning (Tian et al., 2019). This could also explain the dominant role of lipid oxidation in chicken seasoning deterioration (Fig. 1).

## Conclusions

Sensory quality deterioration of chicken seasoning was investigated using quality changes array, GC–MS and descriptive sensory analysis to approach an evaluation of the deterioration of chicken essence seasoning. It was found that the determination of indicator was a novel approach to monitor oil oxidation. They played an important role as TOTOX or POV in the oxidation assessment of lipid. In addition, good relationship between TOTOX and sensory evaluation was found. Moreover, a total of 55 compounds were identified in the chicken seasoning. On a quantitative level, aldehydes were main volatile compounds identified by GC–MS related to the deterioration of chicken essence seasoning. The correlation between unfriendly aldehyde flavor from oil oxidation and sensory attributes was determined using PLSR and PCC. There was a decent compatibility between quality changes assess, descriptive sensory analysis and GC–MS data, indicating that the TOTOX, POV and aldehyde content (especially for hexanal) has the effectiveness indicators to evaluate the quality deterioration of chicken seasoning. These results are promising in terms of the application of the chemical analyses as an objective measurement for conventional sensory evaluations or electronic tongue of the deterioration of chicken seasoning. Moreover, this finding could therefore provide an approach to evaluate quantically the shelf life of chicken seasoning and others food processing applications.

## CRediT authorship contribution statement

**Hao-Yu Xu:** Data curation, Formal analysis, Writing – original draft. **Xiao-Wei Chen:** Conceptualization, Methodology, Writing – review & editing, Supervision. **Jun Li:** Conceptualization, Methodology, Software, Validation. **Yan-Lan Bi:** Visualization, Supervision, Investigation, Project administration, Funding acquisition.

## Declaration of Competing Interest

The authors declare that they have no known competing financial interests or personal relationships that could have appeared to influence the work reported in this paper.

## Data Availability

Data will be made available on request.
